# World‐wide impacts of climate change and nitrogen deposition on vegetation structure, composition, and functioning of shrublands

**DOI:** 10.1111/nph.70235

**Published:** 2025-05-28

**Authors:** Daijun Liu, Chao Zhang, Romà Ogaya, Nezha Acil, Thomas A. M. Pugh, Xavier Domene, Xiwen Zhang, Yunting Fang, Xiaohong Yang, Franz Essl, Stefan Dullinger, Josep Peñuelas

**Affiliations:** ^1^ Department of Botany and Biodiversity Research University of Vienna Rennweg 14 Vienna 1030 Austria; ^2^ Global Ecology Unit CREAF‐CEAB‐CSIC‐Universitat Autònoma de Barcelona Cerdanyola del Vallés (Catalonia) E‐08193 Spain; ^3^ CREAF Cerdanyola del Vallès (Catalonia) E‐08193 Spain; ^4^ Optics of Photosynthesis Laboratory, Institute for Atmospheric and Earth System Research (INAR)/Forest Sciences, Viikki Plant Science Centre University of Helsinki Helsinki 00014 Finland; ^5^ Institute for Environmental Futures, School of Geography, Geology and the Environment, Centre for Landscape and Climate Research University of Leicester Leicester LE1 7RH UK; ^6^ National Centre for Earth Observation University of Leicester Space Park Leicester, 92 Corporation Road Leicester LE4 5SP UK; ^7^ Department of Physical Geography and Ecosystem Science Lund University Lund 22362 Sweden; ^8^ School of Geography, Earth and Environmental Sciences University of Birmingham Birmingham B15 2TT UK; ^9^ Birmingham Institute of Forest Research University of Birmingham Birmingham B15 2TT UK; ^10^ School of Architecture and Urban Planning Guangdong University of Technology Guangzhou 510090 China; ^11^ CAS Key Laboratory of Forest Ecology and Silviculture, Institute of Applied Ecology Chinese Academy of Sciences Shenyang 110164 China; ^12^ Key Laboratory of Horticulture Science for Southern Mountainous Regions, Ministry of Education Southwest University Chongqing 400716 China

**Keywords:** biomass accumulation, climate change, ecological sensitivity, eutrophication, field experiments, shrubland ecosystems, species diversity

## Abstract

Environmental changes and their effects are among the most pressing topics of today's ecological research. Shrublands, although widespread across the globe, remain understudied in this respect. We conducted a global meta‐analysis of 81 shrubland sites subjected to experimental warming, shifts in precipitation (e.g. increased precipitation and drought), and nitrogen addition to quantify seven types of vegetation responses, including density and cover, species diversity, shrub proportion, and ecosystem functions. Our results indicated that the magnitude of responses varied depending on the vegetation metrics and treatment conditions. Specifically, aboveground biomass (AGB) was most sensitive to warming, increased precipitation, and nitrogen addition, while density was most responsive to drought treatment. Short‐term treatments (1–5 yr) generally elicited stronger responses than long‐term ones (> 5 yr), particularly under drought. High sensitivity to changes in climate and nitrogen addition was observed at extremely arid sites (aridity index < 0.2), and water availability strongly mediated sensitivity variation. Surprisingly, many vegetation metrics revealed no association between sensitivity variability and site water availability. Our research offers a global perspective on shrubland vegetation responses to environmental changes, highlighting the importance of water availability in sustaining shrubland biodiversity and functioning under future conditions.

## 概括

环境变化及其影响是当今生态学研究中最为紧迫的议题之一。灌木丛生态系统虽广泛分布于全球，但其变化研究仍相对不足。我们开展一项全球范围的荟萃分析，基于81个灌丛地的增温、降水改变(如降水增加和干旱)以及氮添加实验，以量化包括植被密度与覆盖度、物种多样性、灌木率和生态系统功能等7类响应变量。 研究结果表明植被响应的强度因指标类型和实验条件的不同而异。特别是，地上生物量对增温、增加降水和氮添加最为敏感，然而植被密度对干旱实验的响应最为显著。短期实验(1–5年)通常引发的响应强度高于长期实验(> 5年)，在干旱条件下尤其明显。在极端干旱站点(干旱指数 < 0.2)，对气候变化和氮添加的高度敏感性尤为明显， 并且水分可用性在敏感性变化中起到了关键调节作用。令人意外的是，多项植被指标并未显示出敏感性变化与水分可用性有关联。 我们的研究提供了全球视角的灌丛植被对环境变化响应，强调了水分可用性在未来情境下维持灌丛多样性和生态功能方面的重要性。.

## Introduction

Shrublands are extensively distributed from tropical to sub‐arctic regions, covering 17.6% of the global land surface (Hansen *et al*., 2000; Eldridge *et al*., 2011). As a transitional ecosystem between grassland and forest, shrublands consist of various lifeforms, including shrubs, forbs, and graminoids, playing a vital role in the conservation of biodiversity and terrestrial carbon storage (Brown *et al*., 2002; Eldridge *et al*., 2011; Xian *et al*., 2015; García Criado *et al*., 2020). Conservation and restoration efforts have been implemented in large areas of shrubland ecosystems in order to slow down and reverse biodiversity loss and ultimately achieve carbon neutrality in the future (de Bello *et al*., 2010; Strassburg *et al*., 2020). However, shrublands are increasingly subjected to global drivers of environmental change, such as rapid warming, changes in precipitation patterns (e.g. drought), and nitrogen deposition (Bobbink *et al*., 2010; Eldridge *et al*., 2011; Greaver *et al*., 2016; Mason *et al*., 2022). Given the magnitude of plausible ecological responses to future climate scenarios (e.g. global warming and precipitation shifts) and – in many regions of the world – increasing nitrogen inputs (Ackerman *et al*., 2019), there is an urgent need to improve our knowledge of shrubland biodiversity and functional changes, not least to ensure the effective design of biodiversity conservation and restoration strategies.

Shrubland responses to changes in climate and nitrogen deposition are highly complex, which makes forecasting their future changes a challenge (Brown *et al*., 2001; Emmett *et al*., 2004; Doblas‐Miranda *et al*., 2017). There are context‐specific variations in the direction and magnitude of shrubland responses to environmental changes. For example, climate warming promotes shrub biomass accumulation and encroachment through increases in growth and productivity in cold‐limited climates such as the sub‐arctic (Elmendorf *et al*., 2015; García Criado *et al*., 2020) and mountain climates (Bayle *et al*., 2019). However, these effects may be counteracted (Myers‐Smith *et al*., 2015) or reduced (West *et al*., 2012; Parra & Moreno, 2018; Baudena *et al*., 2020) at sites where low availability of water and/or nutrients limits growth or excludes particular species. The balance of these effects may depend on whether the species at some sites have already adapted to dry and warm conditions through physiological adaptations such as increased water‐use efficiency (Liu *et al*., 2016) or phenological adjustments such as delayed leaf unfolding and flowering (Peñuelas *et al*., 2004). The impacts of nitrogen inputs on shrubland species diversity and composition tend to be even more ecosystem‐ or site‐specific than those of climate (Bobbink *et al*., 2010; Yuan *et al*., 2017). For instance, higher nitrogen input often results in higher vegetation growth and carbon accumulation (Yuan *et al*., 2017; Mason *et al*., 2022), but also triggers species and compositional shifts in water‐limited ecosystems such as those in the Mediterranean regions (Bobbink *et al*., 2010; Benvenutto‐Vargas & Ochoa‐Hueso, 2020). Moreover, shrubland responses to environmental changes fluctuate over time and are closely related to biotic interactions (i.e. competition and facilitation) and species adaptations (i.e. increased drought resistance) (West *et al*., 2012; Andresen *et al*., 2016; Liu *et al*., 2018b, 2020). Additionally, the combined effects of climate change and nitrogen deposition have a significant impact on shrublands. The interaction of drought and nitrogen deposition can result in decreased vegetation cover and shifts in species composition (Valliere *et al*., 2017). The variability of these responses calls for further research to improve our understanding of how shrublands will change under further climate change and nitrogen input in the decades to come.

Manipulative experiments in natural and semi‐natural shrublands provide a valuable opportunity to study the responses of shrubland ecosystems to climate and nitrogen addition (Beier *et al*., 2012; Liu *et al*., 2017; Yuan *et al*., 2017; Halbritter *et al*., 2020). These experiments can reveal changes in species diversity, composition, biomass accumulation, and carbon fluxes (Wu *et al*., 2011; Estiarte *et al*., 2016; Yuan *et al*., 2017; Halbritter *et al*., 2020). For example, under warmer conditions, the accumulation of AGB increased, and composition shifted toward dominant shrub species (i.e. there was shrub encroachment) in manipulated climatic experiments in tundra and mountain habitats (Harte *et al*., 2015; Ylänne *et al*., 2015; H. Liu *et al*., 2018). Especially, it has been established that there are pronounced negative impacts of experimental drought on species diversity, aboveground net primary productivity, and ecosystem respiration in water‐limited Mediterranean sites (Peñuelas *et al*., 2007; Kröel‐Dulay *et al*., 2015, 2022; Estiarte *et al*., 2016; Reinsch *et al*., 2017). Furthermore, it has been shown that shrubland response to simulated drought is greater in short‐term than in long‐term experiments, partly due to the differences in mitigation potential and adaptive capacity (Liu *et al*., 2018b). Nitrogen addition has been shown to enhance biomass accumulation and productivity in temperate shrublands due to increased nutrient availability for dominant species. However, it has also been reported that nitrogen addition can result in declines in vegetation species diversity and composition (Ochoa‐Hueso *et al*., 2013; Benvenutto‐Vargas & Ochoa‐Hueso, 2020) and alter microbial community dynamics, potentially affecting ecosystem carbon fluxes (Song *et al*., 2019). However, these reported responses of shrub communities to climate and nitrogen manipulations are derived from a limited number of study sites. As a result, how widespread and generic they are across large spatial and temporal scales remains unclear.

A meta‐analysis of multiple shrubland experiments across natural systems in which a range of environmental drivers have been manipulated allows for a space‐for‐time approach to illustrate community responses to environmental change (Wu *et al*., 2011; Song *et al*., 2019; Halbritter *et al*., 2020; Liu *et al*., 2021; Smith *et al*., 2024). So far, few meta‐analyses have upscaled ecological responses of shrublands to environmental drivers across different study sites (Kröel‐Dulay *et al*., 2015; Estiarte *et al*., 2016; Reinsch *et al*., 2017; Li *et al*., 2023). Previous studies have focused on functional responses such as carbon‐cycling (Andresen *et al*., 2016; Estiarte *et al*., 2016; Reinsch *et al*., 2017). The structural changes in vegetation cover, species diversity, and composition are insufficiently reported and are unlikely to covary with changes in AGB and productivity (Yue *et al*., 2020; Korell *et al*., 2021; Liu *et al*., 2021). In addition, most assessments of shrubland responses have failed to consider the magnitude of the simulated environmental changes (Kröel‐Dulay *et al*., 2015; Estiarte *et al*., 2016) as well as the duration of field experiments (Leuzinger *et al*., 2011; Liu *et al*., 2017; D. Liu *et al*., 2018; H. Liu *et al*., 2018). Furthermore, how shrubland responses to these environmental drivers depend on habitat conditions (e.g. background climate) also remains unexplored globally. As already discussed, water availability is considered one of the most crucial mediators of shrubland productivity (Peñuelas *et al*., 2007; Estiarte *et al*., 2016; Reinsch *et al*., 2017; Li *et al*., 2023), composition, and diversity responses to climate manipulations across Europe (Kröel‐Dulay *et al*., 2015). Whether this holds true at a global scale remains unclear.

Here, we synthesized and analyzed the changes in shrubland density and cover, species diversity, composition, and functioning in response to manipulated climate (e.g. increased temperature, precipitation, and drought) and nitrogen addition across 81 globally distributed field experiments. We hypothesized that the magnitude of these vegetation metrics may vary with treatment types, with stronger responses observed for the metrics related to ecosystem functioning. We expected that the shrubland responses would be stronger in short‐term experiments, with a higher sensitivity associated with the local climate (e.g. the benefit effect in treatments will be constrained by water availability). Specifically, our objectives were to (1) evaluate the direction and magnitude of shrubland responses in terms of vegetation density and cover, species diversity, composition, and function metrics to changes in climate and nitrogen availability; (2) test for differences caused by variations in experiment duration; and (3) explore the relationships between shrubland responses and the climate of the study sites. The results provide a global perspective on the responses of shrubland communities to simulated global environmental changes, which will be crucial for implementing conservation and restoration efforts to mitigate negative impacts, especially in these water‐limited and temperature‐sensitive ecosystems.

## Materials and Methods

### Data compilation and extraction

We conducted a systematic literature search for relevant studies. First, we searched the Web of Science using the following single term and the combined terms: (1) “shrub” OR “heath” for ecosystems; (2) “species diversity” OR “biomass” OR “cover” OR “aboveground net primary production” OR “ANPP” for vegetation responses; (3) “experiment” OR “treatment” for type of study; and (4) “warm” OR “drought” OR “rainfall” OR “precipitation” OR “irrigation” OR “water” OR “nitrogen” OR “nutrient” for the environmental driver manipulated. Second, we extracted shrubland vegetation responses to climate and nitrogen manipulations from the climatic manipulation data sets (https://droughtnet.weebly.com/) along with the recent peer‐reviewed publications (Komatsu *et al*., 2019; Midolo *et al*., 2019; Song *et al*., 2019; Yue *et al*., 2020; Korell *et al*., 2021; Kröel‐Dulay *et al*., 2022; Smith *et al*., 2024). We selected those plant communities that were dominated by shrubs in the studies. We included studies that were published from 1990 to 2023 because a large number of field experiments, in particular climatic experiments, were conducted starting in the 1990s (Beier *et al*., 2012; Song *et al*., 2019; Liu *et al*., 2021). We identified 5761 studies in the initial search (until December 2023) and selected those that met the criteria for shrubland communities, data availability, and treatment types (details of the selection process in Supporting Information Fig. S1).

We selected the shrubland communities using a broad definition. According to the International Geosphere‐Biosphere Programme‐Data and Information System office (IGBP‐DIS), shrubland is defined as vegetation cover dominated by woody plants that are < 2 m tall (Hansen *et al*., 2000) and which can have either a closed (> 60%) or open canopy (10–60%). Shrublands occur as climax communities in sites that are at the edge of forest habitats or in areas where local environmental conditions (e.g. limitations in water availability or temperature) do not allow forests to develop. They may also be maintained by frequent natural disturbances (e.g. wildfires), or occur as successional stages following anthropogenic disturbances (e.g. grazing, wood harvesting) or land abandonment.

**Fig. 1:**
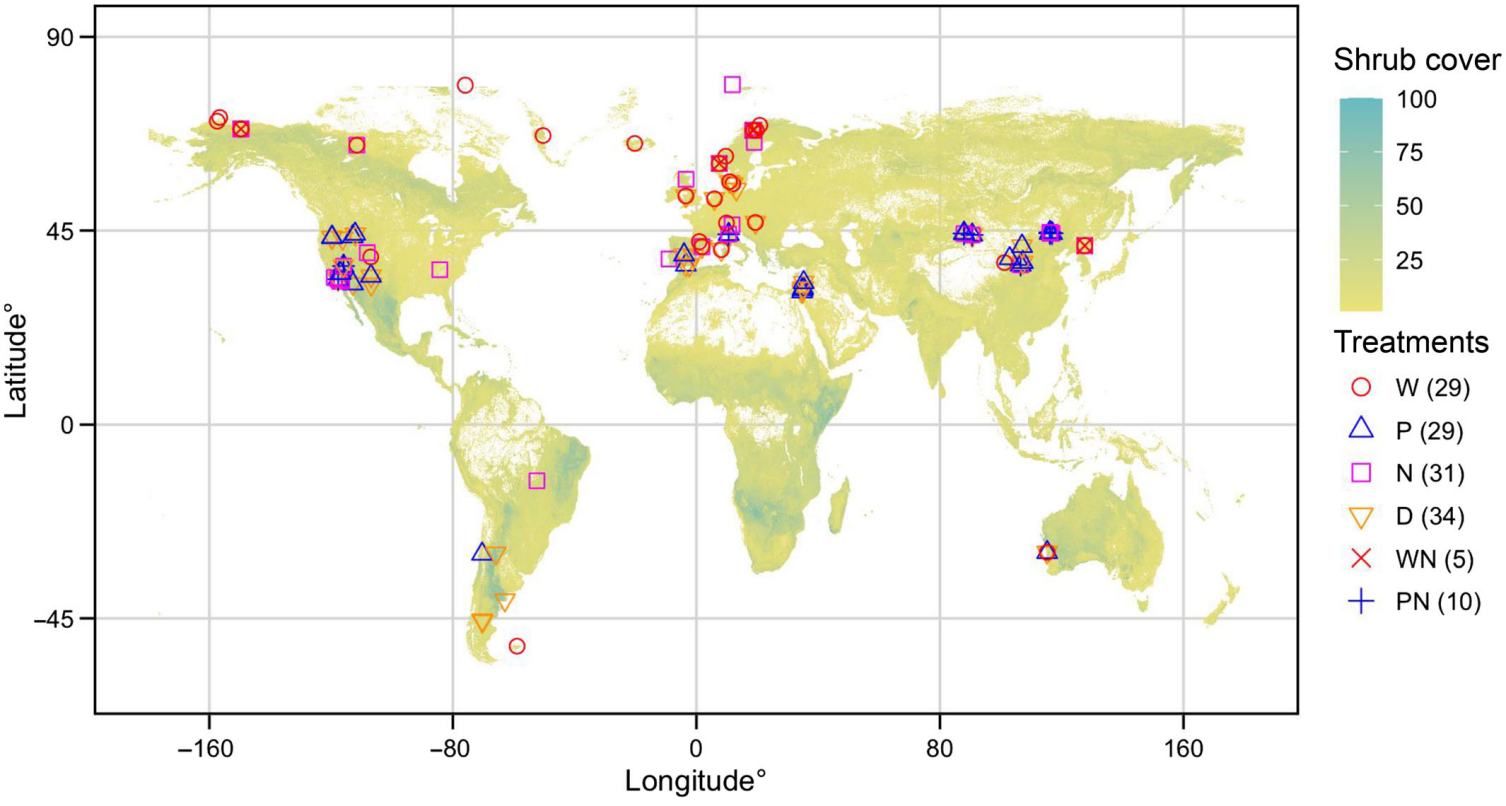
Geographic distribution of the 81 shrubland study sites used in our meta‐analysis. Global shrub cover (fractional cover for shrublands, %) was derived at a 100 × 100 m resolution for the year 2015 (https://lcviewer.vito.be/2015) (Buchhorn *et al*., 2020). Colors represent shrubland communities to experimental warming (W, *n* = 29), increased precipitation (P, *n* = 29), nitrogen addition (N, *n* = 31); drought treatment (D, *n* = 34), warming + nitrogen addition (WN, *n* = 5) and increased precipitation + nitrogen addition (PN, *n* = 10). The *n* indicates the study sites used in the analysis.

As a consequence of the application of these criteria, we constructed a database containing 92 publications representing 81 study sites mainly distributed across North America, Europe, and East Asia in temperate, boreal, and tundra ecoregions (Fig. 1; Table S1). If there were different treatments on a given site, such as winter and summer drought experiments, the corresponding vegetation responses were included separately. Finally, our compilation included study sites on climate warming (*n* = 29), increased precipitation (*n* = 29), nitrogen addition (*n* = 31), and drought (*n* = 34). Additionally, we selected treatment combinations such as warming combined with nitrogen addition (*n* = 5) and increased precipitation combined with nitrogen addition (*n* = 10).

Data relating to shrubland abundance and cover, species diversity, composition, and functions were extracted from the studies. Metrics of community‐level vegetation responses comprised AGB, ANPP, vegetation cover (COV), community density or abundance (DEN), species richness (SP), and species diversity (Shannon diversity index, H) (detailed definitions can be found in Table S2). Shrub encroachment refers to the increases in shrub biomass, density, or cover, which is a symptom of an alteration in ecosystem processes (Stevens *et al*., 2017). Shrub ratio (Ratio_S) was calculated as the proportion of the AGB, density, or cover of the shrub species relative to the values for the entire community (shrubs and herbs) within a plot. If several of those ratios were reported simultaneously, we extracted one of them in our analysis, but considered the ratio of biomass first, then density, and lastly cover. The application of these metrics is explained in Table S2. Whenever appropriate, we extracted vegetation metrics (e.g. AGB, ANPP, COV, DEN, SP, H, and Ratio_S) from the tables and supporting files of the original publications. If the data were not available, we extracted the data from the figures using webplotdigitizer (v.4.5, https://apps.automeris.io/wpd/). Data on mean values, SD, and sample sizes (number of plot replications) of the abovementioned vegetation metrics in treatments and controls were compiled in our meta‐analysis. Each study used only one mean value and SD. If a study provided multiple data points during the study period, the mean values and SD were calculated. The magnitudes of net changes in climate (e.g. warming, increased precipitation, and drought) and nitrogen addition in field experiments were also compiled to quantify the vegetation responses. For experimental warming, year and growing season data were collected separately, and then the magnitudes of climate and nitrogen addition were compiled. Experiment duration was categorized as short‐term (1–5 yr) and long‐term (> 5 yr) (Kröel‐Dulay *et al*., 2015). The geographical location (e.g. latitude and longitude) of the study sites was included to derive details of ambient climates (see below).

### Calculating vegetation responses

In this study, we focused on seven response metrics relating to species diversity, composition, and ecosystem functions. The natural log‐transformed response ratios (RR) were applied to estimate the effect sizes of changes in climate and nitrogen addition. We followed the approach described by Song *et al*. (2019) to calculate the effect size (RR) in field experiments, plus its variance (VI):

(Eqn 1) $$\mathrm{RR}={\text{log}}_{e}\;\left(X_{t}/X_{c}\right)$$

where *X*_*t*_ and *X*_*c*_ represent the treatment and control mean values of plant metrics, respectively.

The variance (VI) is calculated as:

(Eqn 2) $$\mathrm{VI}=\frac{{\mathrm{SD}}_{t}^{2}}{n_{t}X_{t}^{2}}+\frac{{\mathrm{SD}}_{c}^{2}}{n_{t}X_{c}^{2}}$$

where SD_*t*_, *n*_*t*_, and SD_*c*_, *n*_*c*_ represent the SD and sample size of treatment and control plots. Here, the effect size and its variance for each of the seven metrics under each treatment–control pair were calculated separately.

We used the metafor package (v.4.2–0) in R to calculate weighted response ratios and bias‐corrected 95% bootstrap‐confidence intervals using inverse‐variance weighted regressions and random‐effects models for the studies. We transferred the effect sizes of each study into the percentage changes:

(Eqn 3) $$\text{Percentage}\;\left(\%\right)=100\times \left(e^{\mathrm{RR}}-1\right)$$

To assess publication bias, we employed funnel plots to identify asymmetry among the studies. Symmetrical distribution of studies around the mean values in a funnel plot indicates no publication bias. Additionally, we used Egger's regression test to statistically evaluate potential publication bias for each metric in the treatment separately. The results from the funnel plots and Egger's regression tests revealed that some of the metrics exhibited publication biases. Therefore, we applied the Precision Effect Estimate with Standard Error (PEESE) method in our meta‐analysis to account for potential publication bias. This method incorporates the precision of the studies (or the inverse of the variance) as a moderator, providing a more reliable and bias‐corrected effect size in meta‐analyses (Stanley & Doucouliagos, 2014). After correcting for bias using the PEESE method, the publication biases were rare for the metrics (Fig. S2).

The ‘ratio or proportional ecological response per net unit change in drivers’, or otherwise named ‘sensitivity’, has been recommended as an effective approach for the quantification of vegetation changes under environmental change scenarios across multiple study sites (Smith *et al*., 2017; Song *et al*., 2019; Liu *et al*., 2021). We applied this approach to standardise shrubland responses to the different magnitudes of climate and nitrogen manipulations based on the formula from Song *et al*. (2019). For experimental warming, year and growing season data were calculated separately, and then sensitivities were compiled together. Thus, each metric sensitivity to warming and nitrogen addition is expressed as the percentage of response per degree increase in temperature (% + 1°C^−1^) for warming and per gram per year of additional nitrogen (% + 1 g N^−1^ m^−2^ yr^−1^). We multiplied precipitation decrease and increase by 10 mm, respectively, to normalise vegetation responses across all treatments; thus, sensitivity to change in precipitation is expressed as the percentage change (%) of response per 10 mm change in precipitation (% +10 mm^−1^ in increased precipitation and % −10 mm^−1^ in drought, respectively). We did not calculate the sensitivities for the response metrics under treatment combinations due to the low number of studies investigating interactions (*n* = 5 in warming + nitrogen addition and *n* = 10 in increased precipitation + nitrogen addition).

### Ambient climate of the study sites

The site climate information (e.g. MAT and MAP) provided in the literature describes the climate variables across different timeframes, which are not accurate for the comparison. To account for variation in vegetation sensitivity due to study site conditions, we derived the ambient climate for each site from the large‐scale climate datasets. The ambient mean annual temperature was derived from the Worldclim database (https://www.worldclim.org/data/bioclim.html; BIO1) for the period 1970–2000 at *c*. 1 km^2^ resolution (Fick & Hijmans, 2017). The aridity index (AI) for all the study sites was obtained from the Global Aridity and PET Dataset (https://cgiarcsi.community/data/global‐aridity‐and‐pet‐database/) to indicate the level of local water availability, by accounting for rainfall deficits and evapotranspiration. Here, the original AI was used, with higher values indicating more humid conditions.

### Statistical analyses

We (1) assessed the percentage changes in response to treatments, (2) examined the differences emerging from experimental duration, and (3) explored the relationships between percentage changes in response metrics and site background climate. Global patterns of the percentage responses of shrubland vegetation metrics to experimentally manipulated climate and nitrogen addition were tested using the metafor package (v.4.2‐0). Whether these seven selected vegetation metrics responded significantly to climate treatments and/or nitrogen addition was assessed separately for each metric. Wald‐type *z*‐test was used to determine the significance values. Quantitatively, the meta‐regression results deliver the weighted percentage change of metrics per study plus the 95% confidence intervals (CI). To compare the differences between short‐term (1–5 yr) and long‐term (> 5 yr) experiments, experimental duration was treated as a factor in the meta‐regression analysis.

To explore the sensitivity of response metrics to site conditions, we used the ‘rma’ function from the metafor package to fit a meta‐regression model where the site conditions served as a predictor (moderator) of the effect sizes (or standardised sensitivities). In the case of experimental warming, the mean annual temperature and the AI of the study site were used as moderators of these site conditions. We selected the best moderator based on the lowest Akaike information criterion (Symonds & Moussalli, 2011). For nitrogen addition, increased precipitation, and drought treatments, we only used the AI because water availability is known as a strong regulator of vegetation sensitivity (Song *et al*., 2019; Liu *et al*., 2021). All analyses were performed in the R statistical environment (R v.4.2.3).

## Results

### Global patterns of shrubland responses to experimental climate change and nitrogen addition

**Fig. 2:**
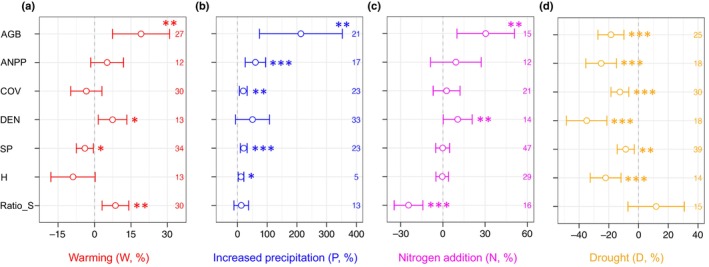
The responses of vegetation metrics to experimental warming (a), increased precipitation (b), nitrogen addition (c), and drought treatment (d) (*, *P* < 0.05; **, *P* < 0.05; ***, *P* < 0.001). Wald‐type *z*‐test is used to determine the significance values. Data are the estimated effect sizes (percentages, %) in response to these treatments, plus their 95% confidence intervals. Dashed lines represent the response to be zero. The values along the right *y*‐axis indicate the number of shrubland communities included in the analysis. AGB, aboveground biomass; ANPP, aboveground net primary production; COV, vegetation cover; DEN, plant density; H, species diversity (Shannon index); Ratio_S, shrub ratio; SP, species richness.

All vegetation metrics were responsive to experimental changes in climate and nitrogen availability, but the responses varied greatly in direction and magnitude (Fig. 2a–d; Table S3). Under warming, most vegetation metrics (4/7) responded positively. Specifically, AGB, vegetation density, and shrub ratio increased significantly under warming (*P* < 0.01, *P* < 0.05, and *P* < 0.05, respectively). Generally, vegetation metrics also responded positively under increased precipitation and nitrogen addition, while the opposite was the case for experimental drought. Indeed, increased precipitation led to significantly higher values for all vegetation metrics, except vegetation density and shrub ratio. Greater availability of nitrogen (nitrogen addition here) significantly increased AGB, vegetation cover, and vegetation density (*P* < 0.01, *P* < 0.05, and *P* < 0.01, respectively) but decreased shrub ratio (*P* < 0.001). By contrast, all metrics were significantly reduced under drought treatments except the shrub ratio, which increased. Most vegetation metrics (except vegetation density) responded negatively to the combination of warming and nitrogen addition (Fig. S3; Table S4), with a significant effect on shrub ratio (*P* < 0.01). However, most responses were positive under increased precipitation combined with nitrogen addition, with significant effects on aboveground net primary productivity and vegetation cover (both *P* < 0.001), whereas the shrub ratio declined (*P* < 0.001).

### Vegetation responses to experimental duration

**Fig. 3:**
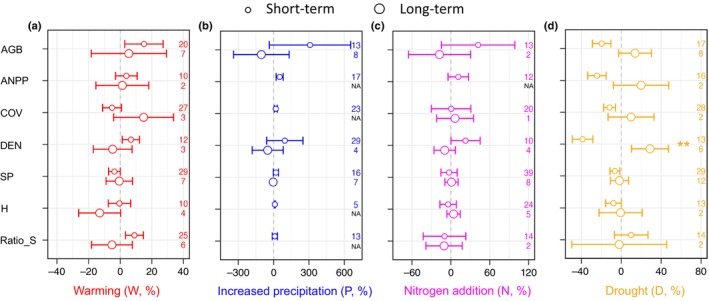
Comparison of responses of vegetation metrics to experimental manipulation of climate (a, b, d) and nitrogen addition (c) at contrasting temporal scales of experimental duration (Short‐term: 1–5 yr, small circle; Long‐term: > 5 yr, large circle). The numbers on the right *y*‐axis indicate the experimental shrub communities included in the analysis. Wald‐type *z*‐test is used to determine the significance values. The asterisks in the figures indicate statistical significance at **, *P* < 0.01. Data are the mean ± 95% confidence intervals. NA in the plots indicates that responses could not be calculated due to a lack of data. AGB, aboveground biomass; ANPP, aboveground net primary production; COV, vegetation cover; DEN, plant density; H, species diversity (Shannon index); Ratio_S, shrub ratio; SP, species richness.

We found a stronger response in the short‐term (1–5 yr) than in the long‐term (> 5 yr) experiments, especially in the climatic treatments (Fig. 3a–d; Table S5). However, as sample sizes were small, statistical power was limited, and hence the difference was only significant in the case of vegetation density (*P* < 0.01), which decreased in the short‐term drought but increased in the long‐term.

### Correlations with site ambient climate

**Fig. 4:**
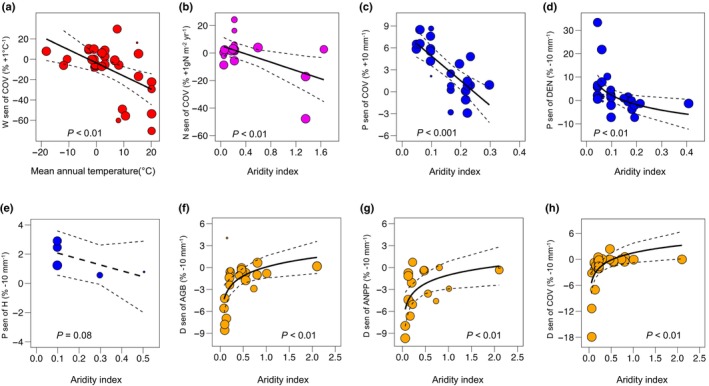
The effect of site conditions, in terms of mean annual temperature and aridity, on the sensitivity of vegetation metrics to experimental treatments. The *y*‐axis describes the sensitivity of a particular metric to a particular treatment; hence (a) refers to warming treatments (W), (b) to nitrogen addition (N), (c–e) to increased precipitation (P), and (f–h) to experimental drought (d). Linear meta‐regressions are shown as black lines, and light dashed lines represent 95% confidence intervals. However, logarithmic regression is used in (d, f–h). Wald‐type *z*‐test is used to determine the significance values. Here, solid regression lines indicate significant correlations (*P* < 0.05), while dashed regression lines indicate marginal significance (*P* < 0.1). AGB, aboveground biomass; ANPP, aboveground net primary production; COV, vegetation cover; DEN, plant density; H, species diversity (Shannon index). The correlation with no significance can be found in Supporting Information Fig. S4.

Meta‐regression analysis indicated that sensitivities of the vegetation metrics were, in one case (vegetation cover under warming), dependent on the mean annual temperature of the study site and, in seven other cases, on the AI (Fig. 4a–h). The sensitivity of vegetation metrics to experimental warming was uncorrelated with aridity and mean annual temperature (Fig. S4a–f), except for vegetation cover (*P* < 0.01; Fig. 4a). Generally, vegetation cover responded positively to experimental warming at cold‐temperature sites (MAT < 5°C) but negatively at warm sites (MAT > 15°C). The sensitivity of vegetation metrics to nitrogen addition was uncorrelated with aridity, except for the case of vegetation cover, which decreased more in drier site conditions (*P* < 0.01). Under increased precipitation, greater sensitivities (or positive) were observed in these arid sites (AI < 0.2) for vegetation metrics (Figs 4c–e, S4g–j). In particular, the sensitivity of vegetation cover, density, and species diversity to additional precipitation tended to shift from positive to negative with decreasing aridity (*P* < 0.001, *P* < 0.05, and *P* < 0.1, respectively). Under drought treatment, the sensitivity of AGB, ANPP, and vegetation cover showed a more negative response in arid sites (AI < 0.5). The trends increased in more humid sites (*P* < 0.05, *P* < 0.05, and *P* < 0.1, respectively), whereas no correlations were observed for the other vegetation metrics (Fig. S4q–t).

## Discussion

Our results reveal that multiple shrubland properties, such as vegetation cover and density, species richness and diversity, and shrub proportion, as well as ecological functions such as productivity, are sensitive to changes in climate and nitrogen availability. In several cases, the magnitude and even the direction of these responses depended on the peculiarities of local climatic conditions, in particular on the level of aridity and, to a lesser degree, on the mean annual temperature of a site. Generally, responses to alteration in precipitation were greater in water‐limited sites, and their changes were strongly associated with local water availability. However, many metrics showed no association with any local climate mediator, indicating that their responses to climatic manipulation and nitrogen addition did not vary across climate gradients. Please note that the local climate data were extracted from the WorldClim datasets (*c*. 1 km^2^ spatial resolution) and may not hence accurately represent the real conditions on the study sites. The association could be improved by using climate information that is specific to the study sites.

### World‐wide shrubland responses to changes in climate and nitrogen addition

Our global meta‐analysis includes the responses of seven shrubland vegetation metrics, showing that shrublands respond in specific ways to changes in climate and nitrogen input. We find that both functional and structural changes are likely to occur in shrubland communities in response to climatic changes and nitrogen addition, reflecting potential characteristics of the future environment. These results differ from previous studies, which reported that shrublands can resist imposed climatic manipulations, as they have already adapted to environmental changes through physiological, demographic, and phenological adjustments (Jump & Peñuelas, 2005; Tielbörger *et al*., 2014). These functional and structural changes appear particularly pronounced under increased water deficit and prolonged and more severe drought conditions that are predicted to occur in many regions in the future (Vicente‐Serrano *et al*., 2012; Smith *et al*., 2024). By contrast, we do not observe significant influences with the interactions of several drivers (except for shrub ratio under warming + nitrogen addition and productivity, vegetation cover and shrub ratio under increased precipitation and nitrogen addition), unlike in other syntheses where synergistic effects of drivers were reported (Komatsu *et al*., 2019; Song *et al*., 2019; Avolio *et al*., 2021). However, this finding is possibly due to the small number of study sites in our dataset where two drivers were experimentally manipulated (five study sites with warming + nitrogen treatments and ten study sites with precipitation + nitrogen treatments).

### Strong responses under short‐term treatments

Taking into account the predicted changes in climate and nitrogen input and the variations in their magnitude, it is important to determine the differences between the duration of field manipulations on changes in species diversity, composition, and functioning (Leuzinger *et al*., 2011; Kröel‐Dulay *et al*., 2015; Andresen *et al*., 2016; Komatsu *et al*., 2019; Liu *et al*., 2022). In our study, we observed generally stronger responses in the short‐term treatments than in the long‐term ones, especially in drought conditions. Under experimental drought, vegetation density was significantly stronger in the short‐term treatment than in the long‐term treatment. These results are partly consistent with the findings from a recent study, which reported that the reduction of ANPP within a single year of extreme drought greatly exceeded previously reported losses for grasslands and shrublands with increasing aridity (Smith *et al*., 2024). We detected that the responses of all vegetation metrics (except shrub ratio) were lower (negative values) in the short‐term drought than in the long‐term (positive values) under experimental drought. This may be consistent with previous studies showing strong treatment effects occurred within shorter time frames (Leuzinger *et al*., 2011; D. Liu *et al*., 2018; H. Liu *et al*., 2018; Liu *et al*., 2022). It should be noted that the number of shrubland communities under long‐term treatment is relatively limited compared to those under short‐term treatments, which may cause an underestimation of the long‐term treatment effect. Possibly, the simplified classification of short‐term (1–5 yr) and long‐term (> 5 yr) used in our study may only partially capture the dependence of effects on the duration of treatments or changes in the environment in general. Future studies should thus focus in more detail on the variability of different metrics of shrubland communities in response to treatment over time.

### Importance of study site water availability on shrubland sensitivities

Shrubland sensitivity to climate and nitrogen addition was generally constrained by site water availability. The model results indicate that the sensitivities of vegetation cover to warming were negatively correlated with the site's mean annual temperature, with more negative values at high temperatures. This result can be attributed to the fact that warming can lead to longer growing seasons, enhanced photosynthesis, and improved nutrient availability for the plant species in cold habitats, while having negative effects in these warm habitats (Kröel‐Dulay *et al*., 2015; Myers‐Smith *et al*., 2015, 2020; D. Liu *et al*., 2018; H. Liu *et al*., 2018). Under nitrogen addition, we only identified the negative correlation between the sensitivity of vegetation cover and the AI. The sensitivities to nitrogen addition for most vegetation metrics showed no correlation with climate mediators of the study sites. Thus, shrubland responses to increased nitrogen availability are site‐dependent and may be influenced by the intricate interplay of nitrogen dynamics (Emmett *et al*., 2004), species composition (Qi *et al*., 2023), and climate variability (Vourlitis *et al*., 2009; Valliere *et al*., 2017; Vourlitis, 2017). However, we found that high (and positive) sensitivities to increased precipitation were observed at low aridity sites. And the sensitivities of vegetation cover, vegetation density, and species diversity were negatively associated with the study site's AI. In such water‐limited habitats, even small increases in water availability can have a significant impact, leading to rapid plant growth and increased diversity as dormant seeds germinate and previously stressed plants thrive (Song *et al*., 2019; Liu *et al*., 2021). These results were similar to reports of negative relationships between biomass accumulation and the study site's water availability in grasslands (Wilcox *et al*., 2017; Liu *et al*., 2021).

Under drought treatments, the responses of AGB, ANPP, and vegetation cover were positively correlated with the increase in aridity values. More negative sensitivities were generally found in the aridity study sites (AI < 0.2), indicating negative effects for these already dry sites to further reduction of water availability (Báez *et al*., 2013; Williams, 2014; Smith *et al*., 2024). This is consistent with previous studies in water‐limited ecosystems, which demonstrated limitations on photosynthesis (Prieto *et al*., 2009b; Liu *et al*., 2016), declines in biomass storage (Prieto *et al*., 2009a; Estiarte *et al*., 2016; Reinsch *et al*., 2017; Smith *et al*., 2024), and losses of species diversity and abundance (Lloret *et al*., 2004; Liu *et al*., 2017, 2022) under drought treatments in the (semi‐)arid areas. By contrast, vegetation density, species richness, and diversity showed less consistent responses (positive and negative) in these water‐limited study sites, and these responses were generally unrelated to the AI. These results contrast with the findings in global grassland communities, for which there is a positive relationship between species diversity and site water availability (Liu *et al*., 2021). Further study is hence necessary to understand the relationships between shrubland structure, species richness, diversity, and drought along climatic gradients.

### Shrub encroachment

Our results show that the shrub ratio was responsive to changes in climate and nitrogen addition. A higher proportion of shrubs was found in the warming treatment, increased precipitation, and drought treatments. This finding echoes a large number of studies reporting and discussing that global environmental changes could accelerate shrub encroachment (e.g. increase the ratio of shrub AGB, density, or abundance and cover) (Knapp *et al*., 2008; Eldridge *et al*., 2011; Stevens *et al*., 2017; Nunes *et al*., 2019; Rolo & Moreno, 2019; García Criado *et al*., 2020). By contrast, we found a lower shrub ratio under nitrogen addition, an effect consistent across almost all of the experimental shrubland communities (15 out of 16) included in our study. In addition, we found that the sensitivity of the shrub ratio to nitrogen addition and even climate manipulation had no relationship with the site's background temperature and water availability. We thus assume that this latter result arises from different ecological strategies of the shrub species, with some obtaining benefits, while others may lose dominance under the various treatments (West *et al*., 2012; Liu *et al*., 2017; D. Liu *et al*., 2018; H. Liu *et al*., 2018; Rolo & Moreno, 2019). Moreover, our meta‐analysis is based on a limited number of studies; in particular, precipitation and nitrogen addition treatments are relatively rare. This phenomenon of shrub encroachment may vary greatly with global environmental drivers and may also be associated with local mediators, such as the site's background climate, soil moisture, and nutrient properties.

### Implications for the fate of shrublands under future environmental change

Our results from experimental manipulations indicate that shrubland species diversity, composition, and functioning are sensitive to warming, shifts in precipitation, and nitrogen addition. As global environmental changes will persist or intensify in the coming decades, shrubland ecosystems may experience significant transformations, with profound impacts on species diversity and composition, carbon storage, and shrubland resilience. The expansion and distribution of shrublands are likely to accelerate as increasing water stress drives widespread episodes of forest die‐offs or transition into shrubland, particularly in water‐limited regions such as Mediterranean ecosystems (McDowell *et al*., 2020; Hammond *et al*., 2022). Additionally, future environmental changes will heighten the extinction risks for species in shrublands, leading to reductions in species diversity, community stability, and carbon storage capacity. Furthermore, projected climate extremes, including severe drought and heatwaves, are expected to diminish shrubland resilience (Smith *et al*., 2024), especially when combined with human activities such as land‐use changes.

We acknowledge the potential limitations of our study and offer four perspectives for future research on field manipulations in shrubland ecosystems. First, conducting field experiments in diverse regions can improve our understanding of shrubland structural and functional dynamics in response to future environmental changes. Currently, most field climate‐change and nitrogen‐addition experiments in shrubland ecosystems are primarily distributed across Northern Hemisphere regions (e.g. USA and Europe), leaving large areas of the Southern Hemisphere underrepresented. Future field manipulations in shrublands should consider geographic distribution to optimize ecological insights, as regions vary in water availability, soil types, and biodiversity. Second, there is an urgent need for experiments that simulate multiple environmental drivers simultaneously, such as increased warming and drought. Only a limited number of field experiments have examined the complex interaction among two or more environmental factors, which fails to elucidate the complex interplay between different environmental drivers. Third, it is essential to design field experiments that explore the effects of extreme events (e.g. severe droughts) and their variability (e.g. timing, frequency, and intensity) on shrublands. To date, most climate manipulation experiments, even those involving extreme drought treatments, have failed to accurately reflect the severe impacts (e.g. on AGB) caused by experimental droughts compared to recent natural extreme events (Kröel‐Dulay *et al*., 2022). Furthermore, the potential tipping points triggered by extreme events have not been adequately identified, which may lead to an underestimation of severe negative impacts, such as substantial species losses or the collapse of critical ecosystem functions. Fourth, a comprehensive evaluation of shrubland structural and functional responses to environmental changes is essential. Systematic analyses and modeling approaches should incorporate a diverse range of ecosystem responses, including both aboveground and belowground metrics, to experimentally manipulated environmental factors. In particular, metrics such as soil respiration, soil organic matter, and nutrient cycling should be included to capture ecosystem changes more comprehensively. Furthermore, detailed information regarding experimental approaches, such as the magnitude of experimental drivers, timing of experiments, and plot size, as well as local conditions such as soil types, nutrient content, and geographical information of the study site, is necessary for accurate assessments of shrubland dynamics under environmental change.

## Competing interests

None declared.

## Author contributions

DL and CZ developed the research ideas, collected and analyzed the data. JP conceived the ideas and extracted soil moisture information. DL and CZ wrote the manuscript with contributions from all co‐authors (RO, NA, TP, XD, XZ, YF, XY, FZ, SD). DL and CZ contributed equally to this work.

## Disclaimer

The New Phytologist Foundation remains neutral with regard to jurisdictional claims in maps and in any institutional affiliations.

## Supporting information


**Fig. S1** Flow diagram illustrating the shrubland studies included in our research.
**Fig. S2** Funnel plots and Egger's regression results for each vegetation metric in response to manipulated climate change and nitrogen addition.
**Fig. S3** Responses of vegetation metrics to the interactions of warming + nitrogen addition (WN) and increased precipitation + nitrogen addition (PN).
**Fig. S4** Relationships between vegetation sensitivity responses to changes in climate and nitrogen addition and the aridity index of the study sites.
**Table S1** List of references of the published studies used in this meta‐analysis.
**Table S2** Definitions of vegetation metrics used in our study.
**Table S3** The percentage responses of vegetation metrics to changes in climate and nitrogen addition.
**Table S4** The percentage responses of vegetation metrics to the combination of warming + nitrogen addition and increased precipitation + nitrogen.
**Table S5** Comparison of the percentage responses of vegetation metrics to changes in climate and nitrogen addition over short‐term (1–5 yr) and long‐term (> 5 yr).
Please note: Wiley is not responsible for the content or functionality of any Supporting Information supplied by the authors. Any queries (other than missing material) should be directed to the *New Phytologist* Central Office.

## Data Availability

The data and R code that support the findings of this study are openly available in Zenodo at https://zenodo.org/uploads/15340864, reference number (doi: 10.5281/zenodo.15340864).
