# Supplementary material for: World‐wide impacts of climate change and nitrogen deposition on vegetation structure, composition, and functioning of shrublands
Source: New Phytol. 2025 May 28;247(3):1117–28. doi: 10.1111/nph.70235 (PMC12222930; doi:10.1111/nph.70235)
Supplement: Supplementary file 1 — Fig. S1 Flow diagram illustrating the shrubland studies included in our research. Fig. S2 Funnel plots and Egger's regression results for each vegetation metric in response to manipulated climate change and nitrogen addition. Fig. S3 Responses of vegetation metrics to the interactions of warming + nitrogen addition (WN) and increased precipitation + nitrogen addition (PN). Fig. S4 Relationships between vegetation sensitivity responses to changes in climate and nitrogen addition and the aridity index of the study sites. Table S1 List of references of the published studies used in this meta‐analysis. Table S2 Definitions of vegetation metrics used in our study. Table S3 The percentage responses of vegetation metrics to changes in climate and nitrogen addition. Table S4 The percentage responses of vegetation metrics to the combination of warming + nitrogen addition and increased precipitation + nitrogen. Table S5 Comparison of the percentage responses of vegetation metrics to changes in climate and nitrogen addition over short‐term (1–5 yr) and long‐term (> 5 yr). Please note: Wiley is not responsible for the content or functionality of any Supporting Information supplied by the authors. Any queries (other than missing material) should be directed to the New Phytologist Central Office. [file NPH-247-1117-s001.pdf]

New Phytologist Supporting Information

Article title: **Worldwide impacts of climate change and nitrogen deposition on vegetation structure, composition and functioning of shrublands**

Authors: Daijun Liu[1,2,3#*], Chao Zhang[4#], Romà Ogaya[2,3], Nezha Acil[5,6], Thomas A. M. Pugh [7,8,9], Xavier Domene[2,3], Xiwen Zhang[10], Yunting Fang[11], Xiaohong Yang[12], Franz Essl[1], Stefan Dullinger[1], Josep Peñuelas[2,3]

Article acceptance date: 27 April 2025

The following Supporting Information is available for this article:

Figure S1. The flow diagram illustrates the shrubland studies included in our research.

Figure S2. Funnel plots and Egger's regression results for each vegetation metric in response to manipulated climate change and nitrogen addition.

Figure S3. Responses of vegetation metrics to the interactions of warming + nitrogen addition (WN) and increased precipitation + nitrogen addition (PN).

Figure S4. Relationships between vegetation sensitivity responses to changes in climate and nitrogen addition and the aridity index of the study sites.

Table S1. List of references of the published studies used in this meta-analysis.

Table S2. The definitions of vegetation metrics are used in our study.

Table S3. The percentage responses of vegetation metrics to changes in climate and nitrogen addition.

Table S4. The percentage responses of vegetation metrics to the combination of warming + nitrogen addition and increased precipitation + nitrogen.

Table S5. The percentage responses of vegetation metrics to changes in climate and nitrogen addition over short-term (1-5 years) and long-term (> 5 years) are compared.

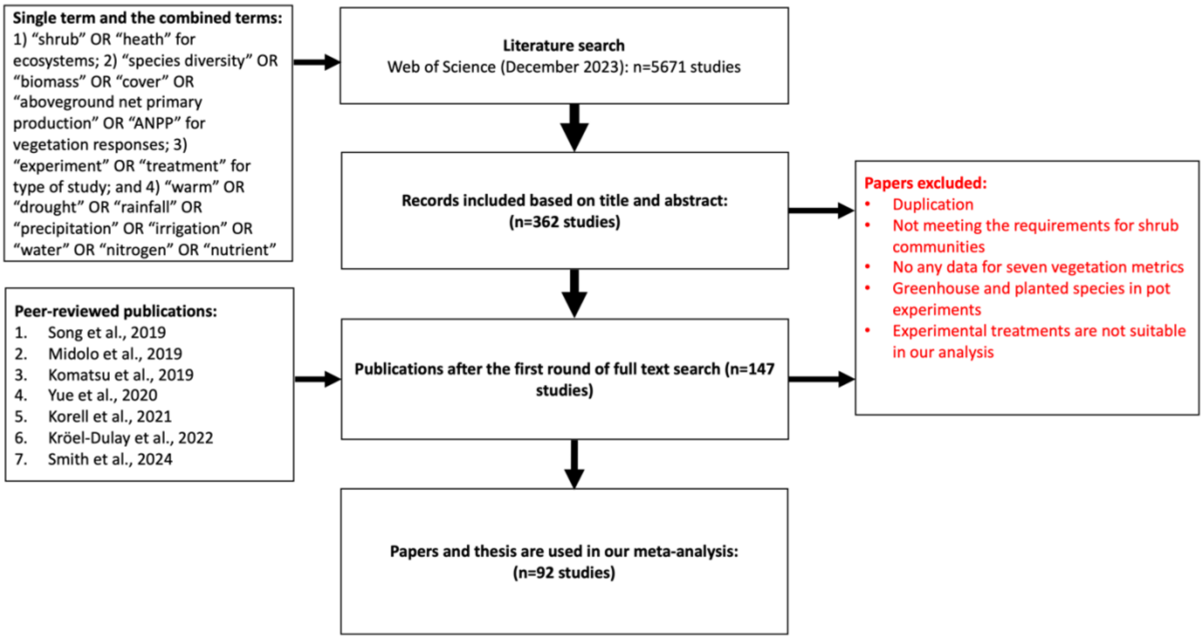

Fig. S1. The flow diagram illustrates the shrubland studies included in our research.

Reference from meta-analysis:

Song, J., Wan, S., Piao, S., Knapp, A. K., Classen, A. T., Vicca, S., ... & Zheng, M. (2019). A meta-analysis of 1,119 manipulative experiments on terrestrial carbon-cycling responses to global change. *Nature ecology & evolution*, *3*(9), 1309-1320.

Midolo, G., Alkemade, R., Schipper, A. M., Benítez-López, A., Perring, M. P., & De Vries, W. (2019). Impacts of nitrogen addition on plant species richness and abundance: A global meta-analysis. *Global ecology and Biogeography*, *28*(3), 398-413.

Komatsu, K. J., Avolio, M. L., Lemoine, N. P., Isbell, F., Grman, E., Houseman, G. R., ... & Zhang, Y. (2019). Global change effects on plant communities are magnified by time and the number of global change factors imposed. *Proceedings of the National Academy of Sciences*, *116*(36), 17867-17873.

Yue, K., Jarvie, S., Senior, A. M., Van Meerbeek, K., Peng, Y., Ni, X., ... & Svenning, J. C. (2020). Changes in plant diversity and its relationship with productivity in response to nitrogen addition, warming and increased rainfall. *Oikos*, *129*(7), 939-952.

Korell, L., Auge, H., Chase, J. M., Harpole, W. S., & Knight, T. M. (2021). Responses of plant diversity to precipitation change are strongest at local spatial scales and in drylands. *Nature communications*, *12*(1), 2489.

Kröel-Dulay, G., Mojzes, A., Szitár, K., Bahn, M., Batáry, P., Beier, C., ... & Peñuelas, J. (2022). Field experiments underestimate aboveground biomass response to drought. Nature Ecology & Evolution, 6(5), 540-545.
Smith, M. D., Wilkins, K. D., Holdrege, M. C., Wilfahrt, P., Collins, S. L., Knapp, A. K., ... & Sun, W. (2024). Extreme drought impacts have been underestimated in grasslands and shrublands globally. *Proceedings of the National Academy of Sciences*, *121*(4), e2309881120.

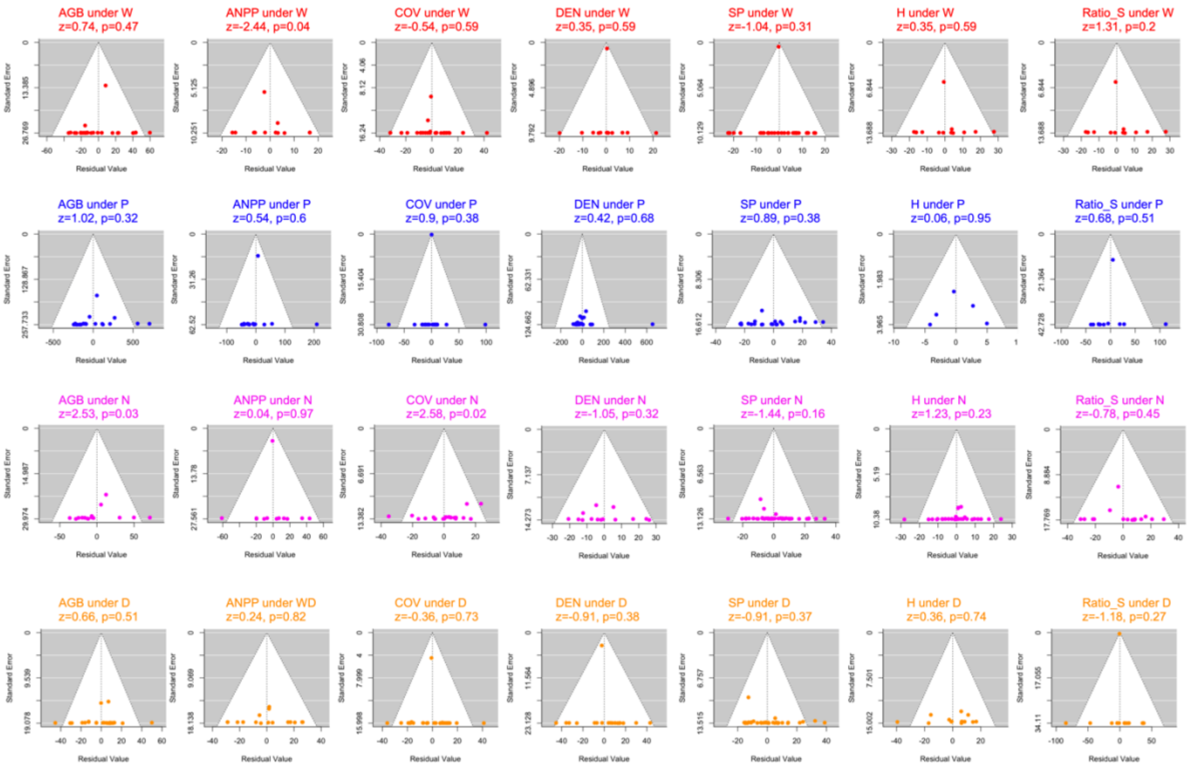

Figure S2. Funnel plots and Egger's regression results for each vegetation metric in response to manipulated climate change and nitrogen addition. The Egger's regression results are shown at the top of each panel (z and p values). The p values > 0.05 indicate the absence of publication bias. The colors indicate the treatments of W: warming; P: increased precipitation; N: nitrogen addition; D: drought. AGB: aboveground biomass; ANPP: aboveground net primary production; COV: vegetation cover; DEN: vegetation density; SP: species richness; H: species diversity (Shannon index); Ratio_S: shrub ratio.

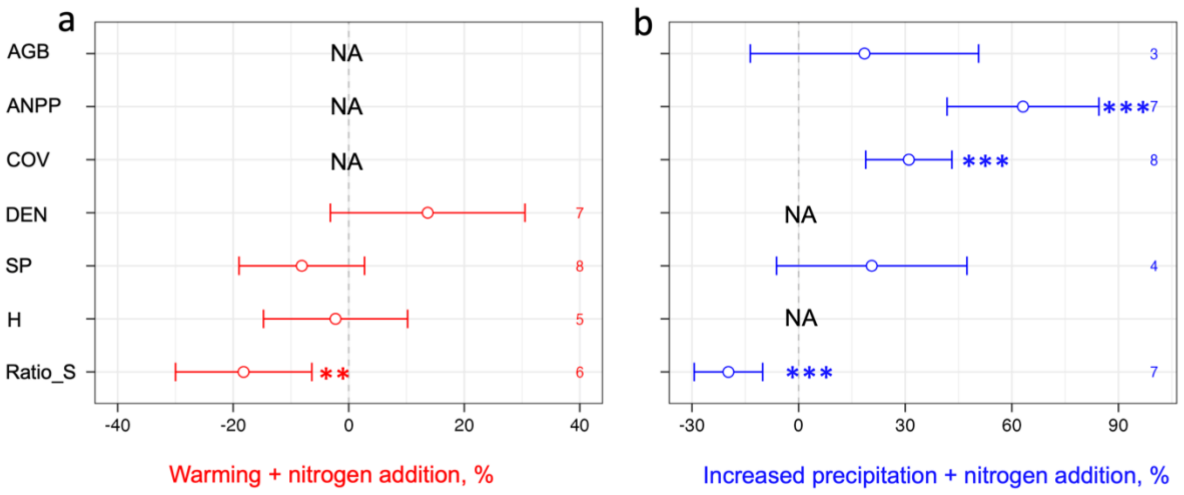

Figure S3. Responses of vegetation metrics to the interactions of warming + nitrogen addition (WN) and increased precipitation + nitrogen addition (PN). Data are means ± the 95% confidence intervals. Dashed lines represent the mean response to be zero. NA in the plots indicate the vegetation metric is lacking data. Wald-type z-test is used to determine the significance values. Statistical significances are shown (**, p < 0.01 and ***, p < 0.001; respectively). The numbers indicate the shrub communities applied into the analysis. AGB: aboveground biomass; ANPP: aboveground net primary production; COV: vegetation cover; DEN: vegetation density; SP: species richness; H: species diversity (Shannon index); Ratio_S: shrub ratio.

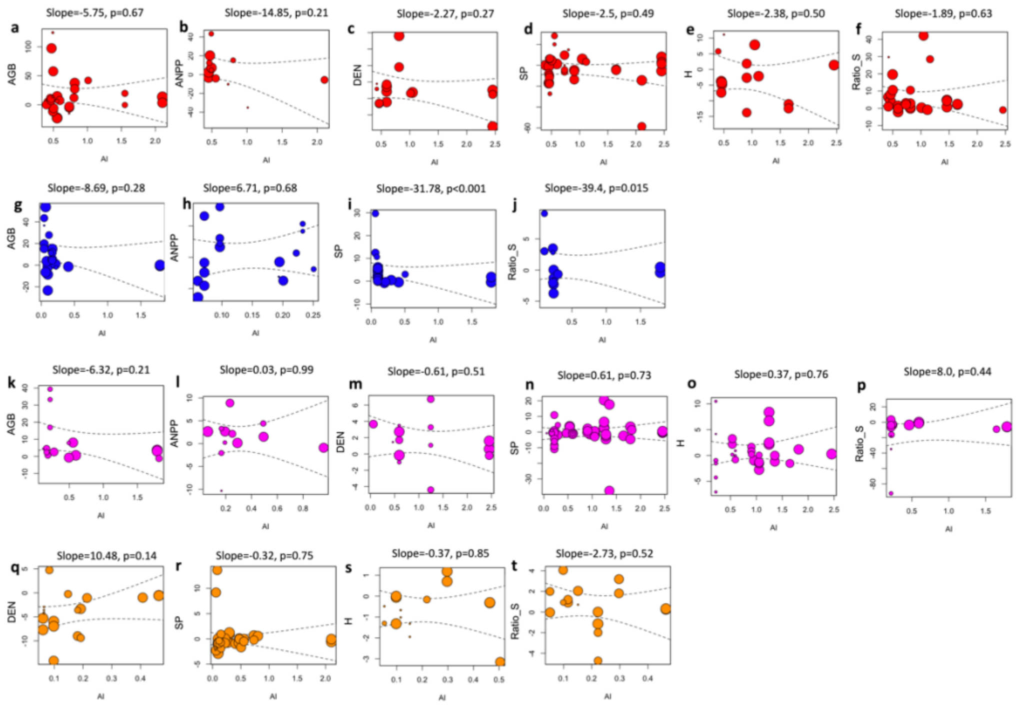

Figure S4. Relationships between vegetation sensitivity responses to changes in climate and nitrogen addition and the aridity index of the study sites. Experiments: warming (a-f), increased precipitation (g-j) and increased nitrogen availability (k-p) as well as drought conditions (q-t). Linear meta-regressions are shown as black lines and light dashed lines representing 95% confidence intervals. Wald-type z-test is used to determine the significant correlation. The slope of the regression lines and the p values are shown at the top of each panel. The colors indicate the treatments of W: warming; P: increased precipitation; N: nitrogen addition; D: drought. AGB: aboveground biomass; ANPP: aboveground net primary production; COV: vegetation cover; DEN: vegetation density; SP: species richness; H: species diversity (Shannon index); Ratio_S: shrub ratio.

Table S1. List of references for the published studies used in this meta-analysis

Number	References
1	Alatalo, J. M., Little, C. J., Jägerbrand, A. K., & Molau, U. (2015). Vascular plant abundance and diversity in an alpine heath under observed and simulated global change. *Scientific reports*, *5*(1), 1-11.
2	Alon, M., & Sternberg, M. (2019). Effects of extreme drought on primary production, species composition and species diversity of a Mediterranean annual plant community. *Journal of Vegetation Science*, *30*(6), 1045-1061.
3	Anadon-Rosell, A., Ninot, J. M., Palacio, S., Grau, O., Nogués, S., Navarro, E., ... & Carrillo, E. (2017). Four years of experimental warming do not modify the interaction between subalpine shrub species. *Oecologia*, *183*(4), 1167-1181.
4	Báez, S., Collins, S. L., Pockman, W. T., Johnson, J. E., & Small, E. E. (2013). Effects of experimental rainfall manipulations on Chihuahuan Desert grassland and shrubland plant communities. *Oecologia*, *172*(4), 1117-1127.
5	Benvenutto-Vargas, V. P., & Ochoa-Hueso, R. (2020). Effects of nitrogen deposition on the spatial pattern of biocrusts and soil microbial activity in a semi-arid Mediterranean shrubland. *Functional Ecology*, *34*(4), 923-937.
6	Bokhorst, S., Convey, P., Huiskes, A., & Aerts, R. (2017). Dwarf shrub and grass vegetation resistant to long-term experimental warming while microarthropod abundance declines on the Falkland Islands. *Austral Ecology*, *42*(8), 984-994.
7	Brancaleoni, L., & Gerdol, R. (2014). Habitat-dependent interactive effects of a heatwave and experimental fertilization on the vegetation of an alpine mire. *Journal of vegetation science*, *25*(2), 427-438.
8	Brancaleoni, L., Gualmini, M., Tomaselli, M., & Gerdol, R. (2007). Responses of subalpine dwarf-shrub heath to irrigation and fertilization. *Journal of Vegetation Science*, *18*(3), 337-344.
9	Campioli, M., Leblans, N., & Michelsen, A. (2012). Twenty-two years of warming, fertilisation and shading of subarctic heath shrubs promote secondary growth and plasticity but not primary growth. *PloS one*, *7*(4), e34842.
10	Carpenter, A. T., Moore, J. C., Redente, E. F., & Stark, J. C. (1990). Plant community dynamics in a semi-arid ecosystem in relation to nutrient addition following a major disturbance. *Plant and Soil*, *126*(1), 91-99.
11	Dawes, M. A., Philipson, C. D., Fonti, P., Bebi, P., Hättenschwiler, S., Hagedorn, F., & Rixen, C. (2015). Soil warming and CO 2 enrichment induce biomass shifts in alpine tree line vegetation. *Global Change Biology*, *21*(5), 2005-2021.
12	De Dato, G., Pellizzaro, G., Cesaraccio, C., Sirca, C., De Angelis, P., Duce, P., ... & Scarascia Mugnozza, G. (2008). Effects of warmer and drier climate conditions on plant composition and biomass production in a Mediterranean shrubland community. *iForest-Biogeosciences and Forestry*, *1*(1), 39.
13	De Dato, G., Pellizzaro, G., Cesaraccio, C., Sirca, C., De Angelis, P., Duce, P., ... & Scarascia-Mugnozza, G. (2006). Effects of warmer and drier climate conditions on plant composition and biomass production in a Mediterranean shrubland community. *Forest Ecology*, *3*, 511-526.
14	DeMarco, J., Mack, M. C., Bret-Harte, M. S., Burton, M., & Shaver, G. R. (2014). Long-term experimental warming and nutrient additions increase productivity in tall deciduous shrub tundra. *Ecosphere*, *5*(6), 1-22.

15 Dias, T., Malveiro, S., Martins-Loução, M. A., Sheppard, L. J., & Cruz, C. (2011). Linking N-driven biodiversity changes with soil N availability in a Mediterranean ecosystem. *Plant and soil*, *341*(1), 125-136.

16 Domínguez, M. T., Sowerby, A., Smith, A. R., Robinson, D. A., Van Baarsel, S., Mills, R. T., ... & Emmett, B. A. (2015). Sustained impact of drought on wet shrublands mediated by soil physical changes. *Biogeochemistry*, *122*, 151-163.

17 Germino, M. J., & Reinhardt, K. (2014). Desert shrub responses to experimental modification of precipitation seasonality and soil depth: relationship to the two-layer hypothesis and ecohydrological niche. *Journal of Ecology*, *102*(4), 989-997.

18 Gough, L., & Hobbie, S. E. (2003). Responses of moist non-acidic arctic tundra to altered environment: productivity, biomass, and species richness. *Oikos*, *103*(1), 204-216.

19 Graglia, E., Jonasson, S., Michelsen, A., Schmidt, I. K., Havström, M., & Gustavsson, L. (2001). Effects of environmental perturbations on abundance of subarctic plants after three, seven and ten years of treatments. *Ecography*, *24*(1), 5-12.

20 Gutiérrez, J. R. (1992). Effects of low water supplementation and nutrient addition on the aboveground biomass production of annual plants in a Chilean coastal desert site. *Oecologia*, *90*, 556-559.

21 Harte, J., Saleska, S. R., & Levy, C. (2015). Convergent ecosystem responses to 23-year ambient and manipulated warming link advancing snowmelt and shrub encroachment to transient and long-term climate–soil carbon feedback. *Global Change Biology*, *21*(6), 2349-2356.

22 Hartley, A. E., Neill, C., Melillo, J. M., Crabtree, R., & Bowles, F. P. (1999). Plant performance and soil nitrogen mineralization in response to simulated climate change in subarctic dwarf shrub heath. *Oikos*, 331-343.

23 Hasvik, Å. (2018). *The effect of long-term experimental warming on lichens and vascular plants in an alpine Dryas heath* (Master's thesis, Norwegian University of Life Sciences, Ås).

24 Henry, H. A., Abedi, M., Alados, C. L., Beard, K. H., Fraser, L. H., Jentsch, A., ... & Yang, X. (2018). Increased soil frost versus summer drought as drivers of plant biomass responses to reduced precipitation: results from a globally coordinated field experiment. *Ecosystems*, *21*(7), 1432-1444.

25 Hollister, R. D., May, J. L., Kremers, K. S., Tweedie, C. E., Oberbauer, S. F., Liebig, J. A., ... & Gregory, J. L. (2015). Warming experiments elucidate the drivers of observed directional changes in tundra vegetation. *Ecology and evolution*, *5*(9), 1881-1895.

26 Huang, G., Li, Y., & Padilla, F. M. (2015). Ephemeral plants mediate responses of ecosystem carbon exchange to increased precipitation in a temperate desert. *Agricultural and Forest Meteorology*, *201*, 141-152.

27 Hudson, J. M., & Henry, G. H. (2010). High Arctic plant community resists 15 years of experimental warming. *Journal of Ecology*, *98*(5), 1035-1041.

28 Jónsdóttir, I. S., Magnússon, B., Gudmundsson, J., Elmarsdóttir, Á., & Hjartarson, H. (2005). Variable sensitivity of plant communities in Iceland to experimental warming. *Global Change Biology*, *11*(4), 553-563.

29 Jordan, S. (2024). *The Interactive Effects of Precipitation and Disturbance on the Functioning of Dryland Ecosystems as Modulated by Mean Annual Precipitation*. Arizona State University.

30 Kimball, S., Goulden, M. L., Suding, K. N., & Parker, S. (2014). Altered water and nitrogen input shifts succession in a southern California coastal sage community. *Ecological Applications*, *24*(6), 1390-1404.

31 Kimball, S., Rath, J., Coffey, J. E., Perea-Vega, M. R., Walsh, M., Fiore, N. M., ... & Allison, S. D. (2024). Long-term drought promotes invasive species by reducing wildfire severity.

32 Klanderud, K., & Totland, Ø. (2005). Simulated climate change altered dominance hierarchies and diversity of an alpine biodiversity hotspot. *Ecology*, *86*(8), 2047-2054.

33 Klein, J. A., Harte, J., & Zhao, X. Q. (2004). Experimental warming causes large and rapid species loss, dampened by simulated grazing, on the Tibetan Plateau. *Ecology Letters*, *7*(12), 1170-1179.

34 Klein, J. A., Harte, J., & Zhao, X. Q. (2007). Experimental warming, not grazing, decreases rangeland quality on the Tibetan Plateau. *Ecological Applications*, *17*(2), 541-557.

35 Kröel-Dulay, G., Ransijn, J., Schmidt, I. K., Beier, C., De Angelis, P., De Dato, G., ... & Penuelas, J. (2015). Increased sensitivity to climate change in disturbed ecosystems. *Nature communications*, *6*(1), 1-7.

36 Liu, D., Estiarte, M., Ogaya, R., Yang, X., & Peñuelas, J. (2017). Shift in community structure in an early-successional Mediterranean shrubland driven by long-term experimental warming and drought and natural extreme droughts. *Global Change Biology*, *23*(10), 4267-4279.

37 Liu, D., Zhang, C., Ogaya, R., Estiarte, M., Zhang, X., Pugh, T. A., & Peñuelas, J. (2022). Delayed and altered post-fire recovery pathways of Mediterranean shrubland under 20-year drought manipulation. *Forest Ecology and Management*, *506*, 119970.

38 Løkken, J. O., Hofgaard, A., Dalen, L., & Hytteborn, H. (2019). Grazing and warming effects on shrub growth and plant species composition in subalpine dry tundra: An experimental approach. *Journal of Vegetation Science*, *30*(4), 698-708.

39 Madan, N. J., Deacon, L. J., & Robinson, C. H. (2007). Greater nitrogen and/or phosphorus availability increase plant species' cover and diversity at a High Arctic polar semidesert. *Polar Biology*, *30*(5), 559-570.

40 Massad, T. J., Balch, J. K., Davidson, E. A., Brando, P. M., Mews, C. L., Porto, P., ... & Trumbore, S. E. (2013). Interactions between repeated fire, nutrients, and insect herbivores affect the recovery of diversity in the southern Amazon. *Oecologia*, *172*(1), 219-229.

41 Matias, L., Castro, J., & Zamora, R. (2011). Soil-nutrient availability under a global-change scenario in a Mediterranean mountain ecosystem. *Global Change Biology*, *17*(4), 1646-1657.

42 McAbee, K., Reinhardt, K., Germino, M. J., & Bosworth, A. (2017). Response of aboveground carbon balance to long-term, experimental enhancements in precipitation seasonality is contingent on plant community type in cold-desert rangelands. *Oecologia*, *183*(3), 861-874.

43 Miranda, J. D. D., Armas, C., Padilla, F. M., & Pugnaire, F. I. (2011). Climatic change and rainfall patterns: effects on semi-arid plant communities of the Iberian Southeast. *Journal of Arid Environments*, *75*(12), 1302-1309.

44 Moloney, K. A., Mudrak, E. L., Fuentes-Ramirez, A., Parag, H., Schat, M., & Holzapfel, C. (2019). Increased fire risk in Mojave and Sonoran shrublands due to exotic species and extreme rainfall events. *Ecosphere*, *10*(2), e02592.

45 Nilsson, M. C., Wardle, D. A., Zackrisson, O., & Jäderlund, A. (2002). Effects of alleviation of ecological stresses on an alpine tundra community over an eight-year period. *Oikos*, *97*(1), 3-17.

46 Olsen, S. L., & Klanderud, K. (2014). Biotic interactions limit species richness in an alpine plant community, especially under experimental warming. *Oikos*, *123*(1), 71-78.

47 Parra, A., & Moreno, J. M. (2018). Drought differentially affects the post-fire dynamics of seeders and resprouters in a Mediterranean shrubland. *Science of the Total Environment*, *626*, 1219-1229.

48 Peñuelas, J., Prieto, P., Beier, C., Cesaraccio, C., De Angelis, P., De Dato, G., ... & Tietema, A. (2007). Response of plant species richness and primary productivity in shrublands along a north–south gradient in Europe to seven years of experimental warming and drought: reductions in primary productivity in the heat and drought year of 2003. *Global Change Biology*, *13*(12), 2563-2581.

49 Post, E. S., Pedersen, C., Wilmers, C. C., & Forchhammer, M. C. (2008). Phenological sequences reveal aggregate life history response to climatic warming. *Ecology*, *89*(2), 363-370.

50 Press, M. C., Potter, J. A., Burke, M. J. W., Callaghan, T. V., & Lee, J. A. (1998). Responses of a subarctic dwarf shrub heath community to simulated environmental change. *Journal of Ecology*, *86*(2), 315-327.

51 Prieto, P., Peñuelas, J., Lloret, F., Llorens, L., & Estiarte, M. (2009). Experimental drought and warming decrease diversity and slow down post-fire succession in a Mediterranean shrubland. *Ecography*, *32*(4), 623-636.

52 Prieto, P., Penuelas, J., Llusia, J., Asensio, D., & Estiarte, M. (2009). Effects of experimental warming and drought on biomass accumulation in a Mediterranean shrubland. *Plant Ecology*, *205*(2), 179-191.

53 Reinsch, S., Koller, E., Sowerby, A., De Dato, G., Estiarte, M., Guidolotti, G., ... & Emmett, B. A. (2017). Shrubland primary production and soil respiration diverge along European climate gradient. *Scientific reports*, *7*(1), 1-7.

54 Richardson, S. J., Press, M. C., Parsons, A. N., & Hartley, S. E. (2002). How do nutrients and warming impact on plant communities and their insect herbivores? A 9-year study from a sub-Arctic heath. *Journal of ecology*, *90*(3), 544-556.

55 Rinnan, R., Stark, S., & Tolvanen, A. (2009). Responses of vegetation and soil microbial communities to warming and simulated herbivory in a subarctic heath. *Journal of Ecology*, *97*(4), 788-800.

56 Salesa, D., Baeza, M. J., Pérez-Ferrándiz, E., & Santana, V. M. (2022). Longer summer seasons after fire induce permanent drought legacy effects on Mediterranean plant communities dominated by obligate seeders. *Science of The Total Environment*, *822*, 153655.

57 She, W., Bai, Y., Zhang, Y., Qin, S., Feng, W., Lai, Z., ... & Miao, C. (2021). Nitrogen-enhanced herbaceous competition threatens woody species persistence in a desert ecosystem. *Plant and Soil*, *460*(1), 333-345.

58 She, W., Bai, Y., Zhang, Y., Qin, S., Jia, X., Feng, W., ... & Qiao, Y. (2020). Nitrogen enrichment suppresses revegetated shrub growth under increased precipitation via herb-induced topsoil water limitation in a desert ecosystem in northern China. *Plant and Soil*, *446*(1), 97-110.

59 She, W., Zhang, Y., Qin, S., Wu, B., & Bai, Y. (2016). Increased precipitation and nitrogen alter shrub architecture in a desert shrubland: implications for primary production. *Frontiers in plant science*, *7*, 1908.

60 Shi, G., Yao, B., Liu, Y., Pan, J., Jiang, S., Wang, Y., ... & Zhou, H. (2021). The effects of long-term warming on arbuscular mycorrhizal fungal communities depend on habitat type on the Qinghai-Tibet Plateau. *Applied Soil Ecology*, *167*, 104030.

61 Sistla, S. A., Moore, J. C., Simpson, R. T., Gough, L., Shaver, G. R., & Schimel, J. P. (2013). Long-term warming restructures Arctic tundra without changing net soil carbon storage. *Nature*, *497*(7451), 615-618.

62 Smith, S., Nowak, R. S., Fenstermaker, L., & Young, M. (2007). *Final Technical Report: Effects of Changing Water and Nitrogen Inputs on a Mojave Desert Ecosystem* (No. DOE/ER/63361-F). Nevada System of Higher Education, Las Vegas, NV; University of Nevada, Las Vegas, NV; University of Nevada, Reno, NV; Desert Research Institute, Las Vegas, NV.

63 Song, M. H., Yu, F. H., Ouyang, H., Cao, G. M., Xu, X. L., & Cornelissen, J. H. (2012). Different inter-annual responses to availability and form of nitrogen explain species coexistence in an alpine meadow community after release from grazing. *Global Change Biology*, *18*(10), 3100-3111.

64 Souza, L., Zelikova, T. J., & Sanders, N. J. (2016). Bottom–up and top–down effects on plant communities: nutrients limit productivity, but insects determine diversity and composition. *Oikos*, *125*(4), 566-575.

65 Sowerby, A., Emmett, B. A., Williams, D., Beier, C., & Evans, C. D. (2010). The response of dissolved organic carbon (DOC) and the ecosystem carbon balance to experimental drought in a temperate shrubland. *European Journal of Soil Science*, *61*(5), 697-709.

66 Sundqvist, M. K., Liu, Z., Giesler, R., & Wardle, D. A. (2014). Plant and microbial responses to nitrogen and phosphorus addition across an elevational gradient in subarctic tundra. *Ecology*, *95*(7), 1819-1835.

67 Svensson, B. M., Carlsson, B. Å., & Melillo, J. M. (2018). Changes in species abundance after seven years of elevated atmospheric CO2 and warming in a Subarctic birch forest understorey, as modified by rodent and moth outbreaks. *PeerJ*, *6*, e4843.

68 Swanson, E. K., Sheley, R. L., & James, J. J. (2021). Shrubs facilitate perennial bunchgrass recruitment in drylands under experimental precipitation change. *Journal of Arid Environments*, *187*, 104432.

69 Tielbörger, K., Bilton, M. C., Metz, J., Kigel, J., Holzapfel, C., Lebrija-Trejos, E., ... & Sternberg, M. (2014). Middle-Eastern plant communities tolerate 9 years of drought in a multi-site climate manipulation experiment. *Nature communications*, *5*(1), 1-9.

70 Toledo, S., Bondaruk, V. F., Yahdjian, L., Oñatibia, G. R., Loydi, A., Alberti, J., ... & Peri, P. L. (2023). Environmental factors regulate soil microbial attributes and their response to drought in rangeland ecosystems. Science of The Total Environment, 892, 164406.

71 Tredennick, A. T., Kleinhesselink, A. R., Taylor, J. B., & Adler, P. B. (2018). Ecosystem functional response across precipitation extremes in a sagebrush steppe. PeerJ, 6, e4485.

72 Valliere, J. M., Irvine, I. C., & Allen, E. B. (2024). Nitrogen deposition suppresses ephemeral post-fire plant diversity. Global Change Biology, 30(1), e17117.

73 Valliere, J. M., Irvine, I. C., Santiago, L., & Allen, E. B. (2017). High N, dry: experimental nitrogen deposition exacerbates native shrub loss and nonnative plant invasion during extreme drought. *Global change biology*, *23*(10), 4333-4345.

74 van Paassen, J. G., Britton, A. J., Mitchell, R. J., Street, L. E., Johnson, D., Coupar, A., & Woodin, S. J. (2020). Legacy effects of nitrogen and phosphorus additions on vegetation and carbon stocks of upland heaths. *New Phytologist*, *228*(1), 226-237.

75 Vourlitis, G. L. (2012). Aboveground net primary production response of semi-arid shrublands to chronic experimental dry-season N input. *Ecosphere*, *3*(3), 1-9.

76 Vourlitis, G. L. (2017). Chronic N enrichment and drought alter plant cover and community composition in a Mediterranean-type semi-arid shrubland. *Oecologia*, *184*(1), 267-277.

77 Vourlitis, G. L., & Pasquini, S. C. (2009). Experimental dry-season N deposition alters species composition in southern Californian mediterranean-type shrublands. *Ecology*, *90*(8), 2183-2189.

78 Vourlitis, G. L., Jaureguy, J., Marin, L., & Rodriguez, C. (2021). Shoot and root biomass production in semi-arid shrublands exposed to long-term experimental N input. *Science of The Total Environment*, *754*, 142204.

79 Vourlitis, G. L., Pasquini, S. C., & Mustard, R. (2009). Effects of dry-season N input on the productivity and N storage of Mediterranean-type shrublands. *Ecosystems*, *12*(3), 473-488.

80 Wen, P., Wang, B., Liu, S., Wu, L., Yue, L., Wu, Y., ... & Chen, D. (2023). Seasonal community stability increased with water addition and shrub removal but reduced with nitrogen addition in semi-arid grassland. *Functional Ecology*, *37*(3), 690-702.

81 Williams, A. (2014). *Climate Change in Southwest Australian Shrublands: Response to Altered Rainfall and Temperature* (Doctoral dissertation, Murdoch University).

82 Yahdjian, L., & Sala, O. E. (2006). Vegetation structure constrains primary production response to water availability in the Patagonian steppe. Ecology, 87(4), 952-962.

83 Yang, X., Xiang, G., Sun, W., Chi, Y., Li, W., Bai, Y., ... & Zheng, S. (2022). Shrub encroachment drives different responses of soil respiration to increased precipitation and N enrichment. *Agricultural and Forest Meteorology*, *325*, 109155.

84 Ylänne, H., Stark, S., & Tolvanen, A. (2015). Vegetation shift from deciduous to evergreen dwarf shrubs in response to selective herbivory offsets carbon losses: evidence from 19 years of warming and simulated herbivory in the subarctic tundra. *Global Change Biology*, *21*(10), 3696-3711.

85 Yu, M. H., He, Y. Y., Zhang, F. C., Ding, G. D., & Wang, C. Y. (2023). Effects of intra-year precipitation variability on shrub community productivity depend on the annual total rainfall. Plant and Soil, 487(1), 499-510.

86 Zamin, T. J., Bret-Harte, M. S., & Grogan, P. (2014). Evergreen shrubs dominate responses to experimental summer warming and fertilization in Canadian mesic low arctic tundra. *Journal of Ecology*, *102*(3), 749-766.

87 Zang, Y. X., Ma, J. Y., Zhou, X. B., Tao, Y., Yin, B. F., & Zhang, Y. M. (2021). Extreme precipitation increases the productivity of a desert ephemeral

plant community in Central Asia, but there is no slope position effect. *Journal of Vegetation Science*, *32*(5), e13077.
Zang, Y. X., Xu, W. X., Wu, K., & Yang, W. K. (2022). Effect of Nitrogen
88 Application on the Sensitivity of Desert Shrub Community Productivity to Precipitation in Central Asia. *Frontiers in Plant Science*, *13*, 916706.
Zhang, C., Willis, C. G., Klein, J. A., Ma, Z., Li, J., Zhou, H., & Zhao, X.
89 (2017). Recovery of plant species diversity during long-term experimental warming of a species-rich alpine meadow community on the Qinghai-Tibet plateau. *Biological Conservation*, *213*, 218-224.
Zong, S., Jin, Y., Xu, J., Wu, Z., He, H., Du, H., & Wang, L. (2016). Nitrogen
90 deposition but not climate warming promotes Deyeuxia angustifolia encroachment in alpine tundra of the Changbai Mountains, Northeast China. *Science of the Total Environment*, *544*, 85-93.
Zuo, X., Cheng, H., Zhao, S., Yue, P., Liu, X., Wang, S., ... & Medina-Roldán,
91 E. (2020). Observational and experimental evidence for the effect of altered precipitation on desert and steppe communities. *Global Ecology and Conservation*, *21*, e00864.
Yu, M. H., He, Y. Y., Zhang, F. C., Ding, G. D., & Wang, C. Y. (2023).
92 Effects of intra-year precipitation variability on shrub community productivity depend on the annual total rainfall. Plant and Soil, 487(1), 499-510.

Table S2. The definitions of vegetation metrics are used in our study

Num	Vegetation metrics (abbreviation)	Definition
1	Aboveground biomass (AGB)	Living vegetation above the soil, including stems, branches, and leaves.
2	Aboveground net primary production (ANPP)	The sum of annual biomass increment of wood parts and the litterfall
3	Vegetation cover (COV)	The relative or percentage of soil which is covered by alive vegetation.
4	Community density (or abundance) (DEN)	The number of hits for alive plant organs or vegetation abundance in a plot.
5	Species richness (SP)	The number of vascular plants within a community.
6	Species diversity (H)	Shannon index is applied here, that is the number of different species in a community (species richness) weighted by the measure of abundance such as number of individuals.
7	Shrub aboveground biomass (AGB_S)	Living vegetation biomass above the soil for the shrub species.
8	Shrub ratio (Ratio_S)	The ratio of shrub aboveground biomass, density or abundance and cover to the community-level values respectively. This is an important vegetation index for shrub encroachment (Stevens et al., 2017).

Note: Stevens, N., Lehmann, C. E., Murphy, B. P., & Durigan, G. (2017). Savanna woody encroachment is widespread across three continents. Global change biology, 23(1), 235-244.

Table S3. The percentage responses of vegetation metrics to changes in climate and nitrogen addition. The responses are the weighted-response ratios and bias-corrected 95% bootstrap-confidence intervals (CI low and CI high) according to inverse-variance weighted regressions and random-effects models from metafor package in R. Wald-type z-test is used to determine the significance values. The numbers indicate the shrub communities are used in the analysis. W: warming; P: increased precipitation; N: nitrogen addition; D: drought; WN: warming + nitrogen addition; PN: increased precipitation + nitrogen addition; AGB: aboveground biomass; ANPP: aboveground net primary production; COV: vegetation cover; DEN: vegetation density; SP: species richness; H: species diversity (Shannon index); Ratio_S: shrub ratio.

**Treat**	**Vegetation metric**	**Responses**	**CI low**	**CI high**	**P value**	**Number**
	Ratio_S	8.65	3.15	14.14	2.03E-03	30
	H	-8.81	-17.94	0.33	5.88E-02	13
	SP	-3.96	-7.49	-0.43	2.81E-02	34
**W**	DEN	7.43	1.52	13.34	1.37E-02	13
	COV	-3.35	-9.78	3.08	3.07E-01	30
	ANPP	5.20	-1.55	11.95	1.31E-01	12
	AGB	19.14	7.39	30.89	1.41E-03	27
	Ratio_S	14.78	-14.61	44.18	3.24E-01	11
	H	11.75	2.27	21.23	1.51E-02	5
	SP	21.05	9.41	32.69	3.93E-04	23
**P**	DEN	50.59	-6.55	107.73	8.27E-02	33
	COV	22.17	8.30	36.04	1.73E-03	21
	ANPP	61.65	24.93	98.37	9.98E-04	16
	AGB	213.37	74.06	352.68	2.68E-03	21
	Ratio_S	-24.40	-34.63	-14.17	2.93E-06	16
	H	-0.44	-4.95	4.07	8.49E-01	29
	SP	-0.14	-5.08	4.80	9.55E-01	47
**N**	DEN	10.54	0.30	20.79	4.37E-02	14
	COV	2.64	-6.89	12.17	5.87E-01	21
	ANPP	9.24	-8.77	27.26	3.15E-01	12
	AGB	30.42	10.00	50.85	3.51E-03	15
	Ratio_S	12.27	-8.03	32.58	2.36E-01	13
	H	-22.10	-32.68	-11.52	4.27E-05	14
	SP	-6.86	-12.27	-1.45	1.29E-02	39
**D**	DEN	-32.00	-43.66	-20.34	7.47E-08	18
	COV	-12.74	-19.21	-6.27	1.13E-04	28
	ANPP	-26.53	-37.40	-15.67	1.70E-06	16
	AGB	-19.00	-27.81	-10.20	2.35E-05	25

Table S4. The percentage responses of vegetation metrics to the combination of warming + nitrogen addition and increased precipitation + nitrogen. The responses are the weighted-response ratios and bias-corrected 95% bootstrap-confidence intervals (CI low and CI high) according to inverse-variance weighted regressions and random-effects models from metafor package in R. Wald-type z-test is used to determine the significance values. The numbers indicate the shrub communities used in the analysis. The NA indicates no data which was not included in the analysis. WN: warming + nitrogen addition; PN: increased precipitation + nitrogen addition; AGB: aboveground biomass; ANPP: aboveground net primary production; COV: vegetation cover; DEN: vegetation density; SP: species richness; H: species diversity (Shannon index); Ratio_S: shrub ratio.

**Treat**	**Vegetation metric**	**Responses**	**CI low**	**CI high**	**P value**	**Number**
	DEN	13.67	-3.17	30.51	0.11160796	7
	H	-2.29	-14.76	10.19	0.71945063	5
	Ratio_S	-18.20	-30.00	-6.40	0.002507	6
**WN**	SP	-8.12	-18.98	2.74	0.14290665	8
	AGB	*NA*	*NA*	*NA*	*NA*	*NA*
	ANPP	*NA*	*NA*	*NA*	*NA*	*NA*
	COV	*NA*	*NA*	*NA*	*NA*	*NA*
	AGB	18.53	-13.59	50.65	2.58E-01	3
	ANPP	63.14	41.77	84.51	6.98E-09	7
	COV	31.02	18.90	43.14	5.22E-07	8
**PN**	Ratio_S	-19.76	-29.39	-10.14	5.70E-05	7
	SP	20.58	-6.21	47.37	1.32E-01	4
	DEN	*NA*	*NA*	*NA*	*NA*	*NA*
	H	*NA*	*NA*	*NA*	*NA*	*NA*

Table S5. The percentage responses of vegetation metrics to changes in climate and nitrogen addition over short-term (1-5 years) and long-term (> 5 years) are compared. The responses are shown as the weighted-response ratios and bias-corrected 95% bootstrap-confidence intervals (CI low and CI high), based on inverse-variance weighted regressions and random-effects models from metafor package in R. Wald-type z-test is used to determine the significance values. The numbers indicate the shrub communities used in the analysis. The NA indicates no data which was not included in the analysis. W: warming; P: increased precipitation; N: nitrogen addition; D: drought; AGB: aboveground biomass; ANPP: aboveground net primary production; COV: vegetation cover; DEN: vegetation density; SP: species richness; H: species diversity (Shannon index); Ratio_S: shrub ratio.

**Treat**	**Metric**	**Duration**	**Responses**	**P values**	**CI low**	**CI high**	**Number**
W	AGB	Short-term	15.0	0.0151	2.9	27.1	20
	AGB	Long-term	5.4	0.6542	-18.4	29.2	7
	ANPP	Short-term	3.9	0.2645	-2.9	10.7	10
	ANPP	Long-term	1.4	0.8737	-15.4	18.1	2
	COV	Short-term	-5.2	0.0906	-11.2	0.8	27
	COV	Long-term	14.8	0.1254	-4.1	33.8	3
	DEN	Short-term	6.8	0.0237	0.9	12.6	12
	DEN	Long-term	1.0	0.9275	-20.1	22.1	1
	H	Short-term	-2.8	0.4518	-10.2	4.5	11
	H	Long-term	-21.5	0.0246	-40.2	-2.8	2
	Ratio_S	Short-term	8.8	0.0024	3.1	14.5	26
	Ratio_S	Long-term	-4.9	0.5335	-20.5	10.6	4
	SP	Short-term	-3.7	0.0490	-7.5	0.0	30
	SP	Long-term	1.0	0.8622	-9.9	11.8	4
P	AGB	Short-term	309.1	0.0794	-36.3	654.4	13
	AGB	Long-term	-103.8	0.3886	-339.7	132.2	8
	ANPP	Short-term	*NA*	*NA*	*NA*	*NA*	*NA*
	ANPP	Long-term	*NA*	*NA*	*NA*	*NA*	*NA*
	COV	Short-term	*NA*	*NA*	*NA*	*NA*	*NA*
	COV	Long-term	*NA*	*NA*	*NA*	*NA*	*NA*
	DEN	Short-term	95.4	0.2250	-58.7	249.4	29
	DEN	Long-term	-50.0	0.4579	-181.9	82.0	4
	H	Short-term	*NA*	*NA*	*NA*	*NA*	*NA*
	H	Long-term	*NA*	*NA*	*NA*	*NA*	*NA*
	Ratio_S	Short-term	*NA*	*NA*	*NA*	*NA*	*NA*
	Ratio_S	Long-term	*NA*	*NA*	*NA*	*NA*	*NA*
	SP	Short-term	19.9	0.0697	-1.6	41.3	16
	SP	Long-term	-3.4	0.6656	-18.9	12.1	7
N	AGB	Short-term	42.0	0.1461	-14.6	98.7	13
	AGB	Long-term	-17.5	0.4737	-65.4	30.4	2
	ANPP	Short-term	*NA*	*NA*	*NA*	*NA*	*NA*
	ANPP	Long-term	*NA*	*NA*	*NA*	*NA*	*NA*

	COV	Short-term	0.5	0.9733	-29.9	31.0	20
	COV	Long-term	6.5	0.6530	-21.9	35.0	1
	DEN	Short-term	22.7	0.0461	0.4	45.1	9
	DEN	Long-term	-9.0	0.2577	-24.5	6.6	5
	H	Short-term	-4.2	0.5105	-16.8	8.4	24
	H	Long-term	4.2	0.4168	-6.0	14.5	5
	Ratio_S	Short-term	-12.5	0.4093	-42.4	17.3	13
	Ratio_S	Long-term	-7.5	0.5372	-31.3	16.3	3
	SP	Short-term	-2.2	0.7290	-14.3	10.0	38
	SP	Long-term	0.6	0.9104	-9.2	10.3	9
	AGB	Short-term	-18.7	p<0.001	-27.8	-9.5	18
	AGB	Long-term	12.2	0.1663	-5.1	29.5	7
	ANPP	Short-term	-25.5	0.0000	-35.1	-15.9	14
	ANPP	Long-term	20.9	0.1316	-6.3	48.0	2
	COV	Short-term	-11.4	0.0005	-17.7	-5.0	27
	COV	Long-term	7.9	0.6448	-25.7	41.5	1
D	DEN	Short-term	-37.1	p<0.001	-47.8	-26.5	14
	DEN	Long-term	29.8	0.0098	7.2	52.4	4
	H	Short-term	*NA*	*NA*	*NA*	*NA*	*NA*
	H	Long-term	*NA*	*NA*	*NA*	*NA*	*NA*
	Ratio_S	Short-term	*NA*	*NA*	*NA*	*NA*	*NA*
	Ratio_S	Long-term	*NA*	*NA*	*NA*	*NA*	*NA*
	SP	Short-term	-6.8	0.0067	-11.7	-1.9	30
	SP	Long-term	1.5	0.7793	-8.7	11.7	9
